# High-speed resistance training vs. low-speed resistance training on body composition and physical function in adults with sarcopenic obesity

**DOI:** 10.1186/s11556-026-00405-1

**Published:** 2026-02-07

**Authors:** Kuo-Jen Hsu, Sheng-Yun Huang, Yi-Hung Liao, Chiao-Nan Chen

**Affiliations:** 1 National Yang Ming Chiao Tung University, Taipei, Taiwan; 2 Department of Physical Therapy, Chung Shan Medical University, Taichung, Taiwan; 3 Physical Therapy Room, Chung Shan Medical University Hospital, Taichung, Taiwan; 4 National Taipei University of Nursing and Health Sciences, Taipei, Taiwan

**Keywords:** Dehydroepiandrosterone sulfate, Muscle power training, Skeletal muscle mass, Testosterone, Traditional resistance training

## Abstract

**Background:**

Sarcopenic obesity (SO) is associated with the highest risk of physical disability. While meta-analyses support resistance training to improve strength and function in this population, the optimal training modality remains unclear. This study compared the effects of high-speed (HSRT) and low-speed resistance training (LSRT) on body composition, hormonal responses, and physical function in adults with SO.

**Methods:**

Seventy-three adults aged > 50 years with sarcopenia and obesity were randomly assigned to the control (*n* = 26), LSRT (*n* = 24), or HSRT (*n* = 23) group. Both LSRT and HSRT groups performed progressive resistance training twice weekly for 16 weeks at 70% 1-RM, differing only in concentric contraction speed. The control group maintained their usual lifestyle throughout the study period. All participants underwent assessments of body composition (by dual-energy X-ray absorption), salivary hormone levels (testosterone, dehydroepiandrosterone sulfate (DHEA-S), and cortisol), and physical function at baseline and after 16 weeks. Physical function was also assessed at mid-term (week 8). Intervention effects were analyzed using two-way repeated-measures ANOVA, with significance set at *p* < 0.05.

**Results:**

Intervention influenced body composition, anabolic hormones, and physical functions of participants (*p* < 0.05). The LSRT group showed a significant increase in total body muscle mass (+ 1.14%), whereas no such changes were observed in the HSRT or control groups. Salivary DHEA-S levels were maintained in the LSRT and HSRT groups but significantly declined in the control group. Compared to the control group, both LSRT and HSRT groups exhibited significant improvements in all physical function measures at both 8 and 16 weeks.

**Conclusions:**

Both LSRT and HSRT improved physical function and may help maintain certain anabolic markers (such as DHEA-S) in adults with SO. A trivial increase in muscle mass was observed only in the LSRT group.

**Trial registration:**

This study was registered with the Thai Clinical Trial Registry (TCTR) under the registration number TCTR20220107001. The registration was first posted on 07 January 2022, and the study commenced on 24 February 2022.

## Introduction

Skeletal muscle mass declines by 1–2% per year after age 50 [1]. When this loss progresses beyond a certain threshold, it is defined as sarcopenia. Several criteria have been proposed to define low muscle mass, with the most common being appendicular skeletal muscle mass (ASM) adjusted by body height (ASM/Ht^2^), body weight (ASM/Wt), or body mass index (ASM/BMI) (Table 1). When sarcopenia coexists with excessive accumulation of body fat, the condition is termed sarcopenic obesity (SO) [2]. Similarly, obesity can be defined using various indicators, such as BMI, waist circumference (WC), and body fat percentage (BF%) **(**Table 1**)**. A 6-year longitudinal study revealed SO exacerbates walking speed decline with aging [3]. Individuals with SO, compared to individuals with sarcopenia alone and obesity alone, had a significantly lower physical function and higher risk of disability [4–6].

**Table 1:** The definition of sarcopenia and obesity

	Reference/Guidelines	Criteria
**Sarcopenia**	EWGSOP2 [70]	ASM/Height^2^ <7.0 kg/m^2^ for men; <5.5 kg/m^2^ for women*
	ESPEN/EASO [71]	ASM/weight < 29.5–32.5% for men; <19.4–25.7% for women*
	AWGS [33, 35]	ASM/Height^2^ <7.0 kg/m^2^ for men ; <5.4 kg/m^2^ for women* ASM/BMI < 0.80 for men; <0.55 for women*
	Lim et al., 2010 [36]	ASM/weight < 29.9% for men; <25.1% for women*
	Studenski et al., 2014 [37]	ASM/BMI < 0.789 for men; <0.512 for women*
**Obesity**	WHO [72]	BMI > 30 kg/m^2^; WC > 102 cm for men; >88 cm for women
	ESPEN/EASO [71]	BF% > 20.21-31% for men; >31.71–42.9% for women
	Ministry of Health and Welfare, Taiwan [38]	BF% > 25% for men; > 30% for women

*AWGS* Asian Working Group for Sarcopenia, *ASM* appendicular skeletal muscle mass, *BF%* body fat percentage, *BMI* body mass index, *EASO* the European Association for the Study of Obesity, *ESPEN* European Society for Clinical Nutrition and Metabolism, *EWGSOP2* European Working Group on Sarcopenia in Older People 2, *WHO* World Health Organization

*ASM and BF% is assessed by dual-energy X-ray absorptiometry

The age-related loss of muscle mass is associated with hormone changes and the imbalance between anabolic and catabolic hormones [7–11]. Testosterone and its precursor dehydroepiandrosterone sulfate (DHEA-S) are primary anabolic hormones that play a key role in maintaining muscle mass [12, 13]. On the contrary, cortisol is a key catabolic hormone involved in muscle breakdown [14]. The testosterone-to-cortisol (T/C) ratio is widely used as an indicator of the anabolic-catabolic balance [15]. Studies have found that individuals with sarcopenia or obesity have lower levels of testosterone and DHEA-S and higher levels of cortisol compared to those without these conditions [16–18]. 8–10 weeks of resistance training have been found to increase resting levels of testosterone and DHEA-S and decrease resting levels of cortisol in young adults [19–21]. Nevertheless, the effects of resistance training on key anabolic and catabolic hormones in individuals with SO remain unclear.

It is well established that resistance training improves physical function and muscle mass in adults with SO [22–24]. Traditionally, low-speed resistance training (LSRT) is employed due to safety concerns. But high-speed resistance training (HSRT) was proposed recently because muscle power is positively correlated with physical function (chair stand test, time up-and-go test, stairs climbing test) [25–27]. Current studies suggest that both high-speed and low-speed resistance training improve physical function in older adults [28–30]. HSRT may be more beneficial for older adults who are sedentary than LSRT [31, 32]. Nevertheless, it remains unclear whether high- and low-speed resistance training have differential effects on physical function and body composition in adults with SO. Thus, this study aimed to investigate the effects of high-speed and low-speed resistance training on body composition, hormones, and physical function in adults with SO.

## Methods

### Study design

This study was a randomized controlled trial. All participants signed the informed consent form before taking any tests or training. Participants were randomly assigned to the control group, the low-speed resistance training (LSRT) group, and the high-speed resistance training (HSRT) group after completing baseline assessments. The randomization process was conducted using a random number generator in Microsoft Excel by an individual who was not involved in the assessment or intervention. The group assignments were concealed until the completion of baseline assessments, ensuring allocation concealment throughout the process. Participants were aware of being assigned to either the control or exercise group. However, those in the exercise group were not informed whether they belonged to the LSRT or HSRT group. All training sessions were conducted under one-on-one supervision, and participants followed the instructor’s guidance. Due to the one-on-one training format, participants in the LSRT and HSRT groups were unaware that their prescribed concentric contraction speeds differed from those of the other group. The assessors were not blinded to group allocation. However, body composition and hormone levels were analyzed using dual X-ray absorption (DXA) and spectrophotometric assay kits, respectively, which minimized the potential for assessor bias. All physical function tests were conducted following standardized procedures to minimize potential bias.

Participants in the control group were asked to maintain their lifestyle for 16 weeks. Participants in the LSRT and HSRT groups attended resistance training twice a week for 16 weeks. All participants underwent body composition examination by DXA (Lunar iDXA, GE Medical System, Madison, WI), did physical function tests, and the resting saliva was collected (for testosterone, DHEA-S, and cortisol analysis) at baseline and after 16 weeks. According to past literature, 8 weeks of resistance training could significantly improve physical function in older adults [28]. Therefore, physical function was also assessed after 8 weeks. This study was approved by the Institutional Review Board of National Yang Ming Chiao Tung University (IRB number: YM109007F) and was registered in Thai Clinical Trial Registration (registry number: TCTR20220107001).

### Participants

A total of 73 participants were recruited in this study. Inclusion criteria included (1) aged > 50 y/o, (2) sarcopenia, and (3) obesity. The inclusion of participants aged over 50 years was based on evidence that skeletal muscle mass declines by approximately 1–2% per year after this age [1]. The 2025 consensus of the Asian Working Group for Sarcopenia (AWGS) also recommends extending the diagnostic criteria for sarcopenia to middle-aged adults (50–64 years) [33]. Moreover, the prevalence of obesity peaks in middle age [34], making this population particularly relevant for studying SO. In this study, sarcopenia was defined by ASM/Ht^2^ < 7.0 kg/m^2^, ASM/Wt < 29.9%, or ASM/BMI < 0.789 for men, ASM/Ht^2^ < 5.4 kg/m^2^, ASM/Wt < 25.1%, or ASM/BMI < 0.512 for women [35–37]. The use of multiple criteria enabled a more comprehensive identification of individuals with sarcopenia, taking into account differences in body size. Obesity was defined by body fat > 25% and 30% for men and women, respectively, according to the definition of obesity used in Taiwan [38]. Exclusion criteria included (1) regular resistance training behavior (> 2 times/week in the past year), (2) women still with menstrual cycles, and (3) neurological diseases or other diseases that prevent individuals from assessments or training.

### Intervention

Participants in the LSRT and HSRT groups performed progressive resistance training at 70% of predicted one repetition maximal (1RM) twice a week for 16 weeks (with at least 48 h between non-consecutive training days). Predicted 1RM was predicted by the 5RM test for each training exercise to prevent injury of participants by the formula: predicted 1RM=5RM×(1་0.025 × 5) [39]. The training consisted of five exercises: chest press, upright row, knee extension, hip abduction, and squat. Except for the squat, which was performed without a machine, the other exercises (chest press, upright row, knee extension, and hip abduction) were conducted using a multifunctional training station (Bravo Advanced, Cybex, USA) where participants performed training exercises in an independent standing position, rather than sitting or relying on a fixed support. For both exercise groups, each exercise was performed for 3 sets of 10 repetitions with 1 min of rest between sets throughout the 16-week training period. This training frequency and volume align with current recommendations for resistance training in older adults [40]. Note that, although current guidelines for older adults recommend 8–10 exercises targeting major muscle groups, five exercises were selected to balance adequate muscle engagement with a feasible and safe training volume, given the sedentary lifestyle, reduced physical capacity, and adherence challenges in this population.

The difference between the two training groups was the speed of concentric contractions. The LSRT group completed the concentric contraction between 3 and 4 s, whereas the HSRT group completed the concentric contraction as fast as possible (within 1 s). The LSRT and HSRT groups had the same eccentric contraction speed (between 3 and 4 s). The metronome was used throughout all training sessions to ensure a consistent tempo among participants. During the first two months (weeks 0–8), training intensity was determined based on the predicted 1RM assessed at baseline. To ensure progressive overload and optimize training effects, the predicted 1RM was re-evaluated at week 8. Training intensity during weeks 9–16 was then determined based on the updated predicted 1RM. The predicted 1RM was reassessed only once, at week 8, based on previous resistance training studies in middle-aged and older adults. When the training duration was less than 12 weeks and the predicted 1RM was used to determine training intensity, interim strength reassessments were generally not performed [41]. In longer interventions, reassessment is typically conducted midway through the program to recalibrate training loads and ensure appropriate progression [42].

### Outcomes variables

#### Body composition and physical function

Body weight, and total and regional (appendicular and trunk) fat and muscle mass were measured using dual-energy X-ray absorptiometry (Lunar iDXA, GE Medical System, Madison, WI).

Physical function of participants was determined by chair stand test (CST), timed up-and-go test (TUG), and stairs climbing test (SCT), which are validated tests for assessing mobility in older adults [43]. In the CST, participants sat on the chair (without armrests) with arms crossed over the chest. When assessors said “start”, participants stood up and sat down repeatedly as fast as possible for 30 s. The times completed in the 30 s were recorded [5]. In the TUG, participants sat on the chair (without armrests). When assessors said “go”, participants stood up from the chair, walked 8 feet, turned around, walked back to the chair, and sat down as fast as possible. The time required to complete the TUG test was recorded [5]. In the SCT, participants stood in front of the stairs. When assessors said “go”, participants climbed the flight of stairs (11 steps, 18 cm/step) as fast as possible without using handrail. The time required to complete the SCT was recorded [44].

#### Salivary testosterone, DHEA-S, and cortisol

Salivary testosterone, DHEA-S, and cortisol levels were determined by spectrophotometric assay kits according to the manufacturer’s instructions (Salimetrics, State College, PA, USA). Salivary testosterone, DHEA-S, and cortisol levels were found to be highly correlated with serum testosterone, DHEA-S, and cortisol levels [45–47]. Saliva samples were collected from participants after a 10-minute rest. To avoid interference, sample collection was scheduled at least 60 min after a major meal and 12 h after alcohol consumption. Participants rinsed their mouths thoroughly with water 10 min prior to sample collection to minimize the impact of acidic or high-sugar foods on assay performance. In addition, to minimize the influence of circadian rhythm, saliva samples were consistently collected at the same time of day both before and after the 16-week intervention. Saliva samples were collected in Salivette^®^ cotton tubes, centrifuged at 1500 g for 15 min, and the supernatant was stored at -20 °C for analysis.

### Sample size calculation

Based on previous studies investigating the effects of resistance training on muscle mass in the elderly, the effect size ranged from 0.2 to 0.8 [28, 48]. The sample size was calculated using G*Power, where the effect size was set at 0.2, the type I error was 0.05, and the type II error was 0.20 (statistical power: 80%). The required sample size was 21 participants per group. At least 23 participants were recruited per group to account for potential dropouts.

### Statistics

Statistical analyses were performed by Statistical Package for the Social Sciences 25.0 (SPSS 25.0, Chicago, USA). Data were presented as mean ± standard error or number (percentage). The normality of the data distribution was assessed using the Shapiro–Wilk test. Differences in baseline characteristics among groups were assessed using one-way analysis of variance (ANOVA) for continuous variables and chi-square tests for categorical variables. Two-way repeated measures ANOVA was used to examine the interaction between groups (control, LSRT, and HSRT) and time (0, 8, and 16 weeks after intervention). The Bonferroni test was used as a post-hoc test. Paired t-tests were used for within-group comparisons. Effect sizes were calculated with Cohen’s *d* and interpreted according to Cohen’s criteria: small (0.2 ≤ d < 0.5 ), medium (0.5 ≤ d < 0.8), and large (d ≥ 0.8) [49]. An intention-to-treat analysis was employed, and missing data were imputed using the mean value of the corresponding group. The significance level was set as 0.05.

## Results

### Basic characteristics of participants

**Fig. 1:**
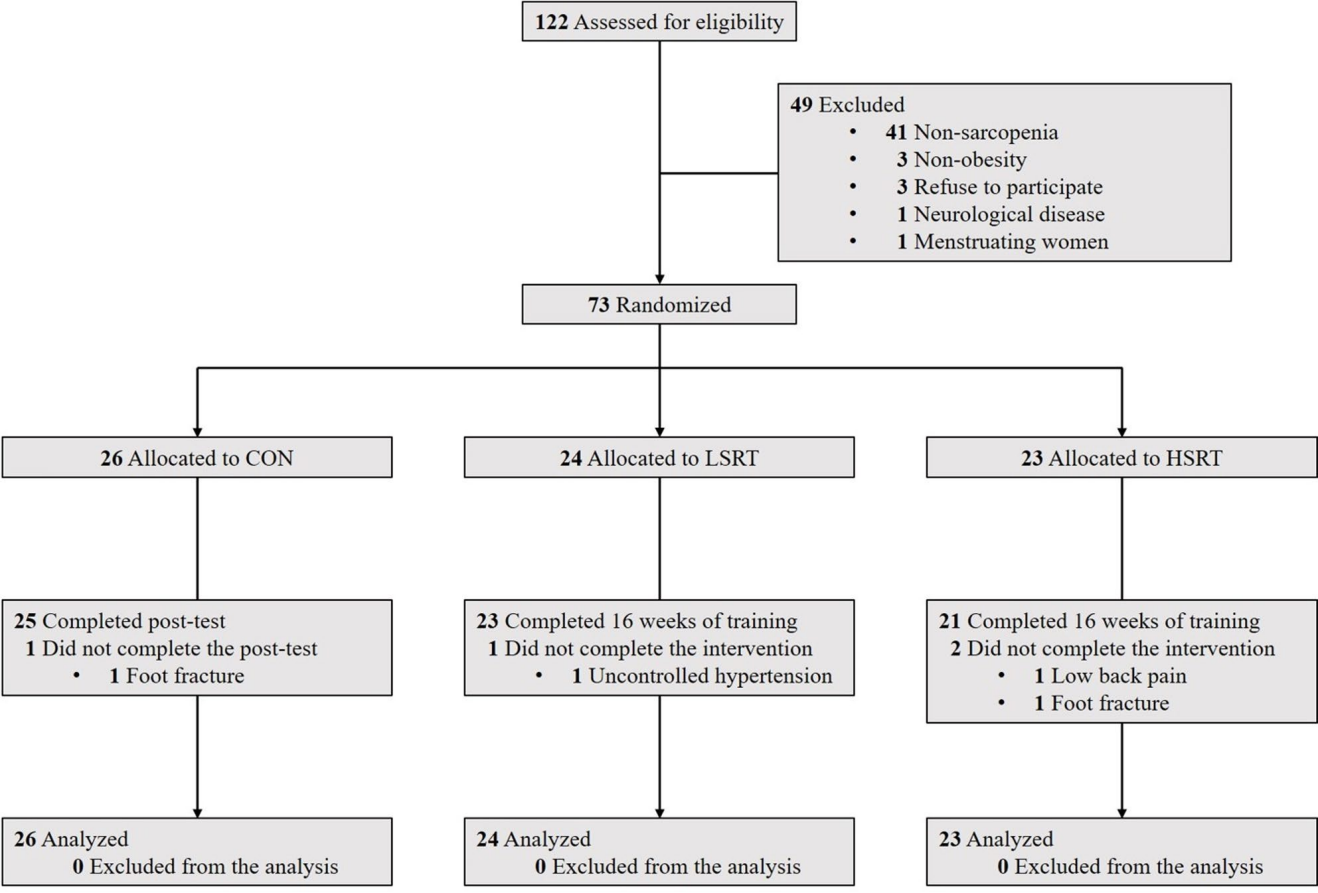
Flow chart of the study. CON: control group; HSRT: high-speed resistance training group; LSRT: low-speed resistance training group

Seventy-three participants (63 women, 10 men) were randomized to the control group (*n* = 26), LSRT group (*n* = 24), and HSRT group (*n* = 23) **(**Fig. 1**)**. The average age of all participants was 65.9 ± 5.8 years old. The average BMI, WC, and percentage of body fat of all participants were 25.1 ± 3.9 kg/m^2^, 85.0 ± 9.8 cm, and 38.6 ± 5.3%, respectively. All participants’ average ASM/Ht^2^, ASM/Wt, and ASM/BMI were 6.2 ± 0.9 kg/m^2^, 24.8 ± 2.0%, and 0.621 ± 0.099, respectively. There were no differences in age, sex, BMI, WC, percentage of body fat, and all skeletal muscle indexes among groups (*p* > 0.05) **(**Table 2**)**.

**Table 2:** Basic characteristics

Age (y/o)	CON (*n* = 26)	LSRT (*n* = 24)	HSRT (*n* = 23)	*p*-value
	64.7 ± 1.2	66.7 ± 1.1	66.3 ± 1.3	0.439
Men (%)	3 (11.5)	3 (12.5)	4 (17.4)	0.820
BMI (kg/m^2^)	26.0 ± 0.7	25.6 ± 0.8	23.6 ± 0.8	0.064
Body fat (%)	39.5 ± 1.0	39.8 ± 1.0	36.5 ± 1.1	0.070
Waist circumference	87.0 ± 1.8	84.9 ± 9.6	83.0 ± 2.2	0.361
ASM/Ht^2^ (kg/m^2^)	6.3 ± 0.2	6.3 ± 0.2	6.1 ± 0.2	0.522
ASM/Wt (%)	25.0 ± 0.5	24.7 ± 0.4	25.9 ± 0.4	0.176
ASM/BMI	0.621 ± 0.022	0.620 ± 0.018	0.647 ± 0.021	0.592
Hypertension (%)	8 (30.8)	9 (37.5)	6 (26.1)	0.698
Type 2 DM (%)	6 (23.1)	4 (16.7)	3 (13.0)	0.647
Dyslipidemia (%)	11 (42.3)	9 (37.5)	7 (30.4)	0.690
CST (times)	19.0 ± 0.8	18.7 ± 1.2	19.2 ± 0.9	0.932
TUG (sec)	5.95 ± 0.18	6.30 ± 0.18	6.03 ± 0.19	0.360
SCT (sec)	5.40 ± 0.29	5.94 ± 0.45	5.51 ± 0.23	0.483

Data were presented as mean ± SE

*ASM* appendicular skeletal muscle mass, *BMI* body mass index, *CON* control group, *CST* chair stand test, *DM* diabetes mellitus, *HSRT* high-speed resistance training group, *Ht* body height, *LSRT* low-speed resistance training group, *SCT* stairs climbing test, *TUG* timed up-and-go test, *Wt* body weight

Regarding the prevalence of chronic diseases, 31.5% of participants (*n* = 23) had diagnosed hypertension, 17.8% (*n* = 13) had type 2 diabetes, and 35.6% (*n* = 26) had dyslipidemia. There were no differences in the prevalence of chronic diseases among groups (*p* > 0.05) **(**Table 2**)**.

### Compliance with the intervention

**Fig. 2:**
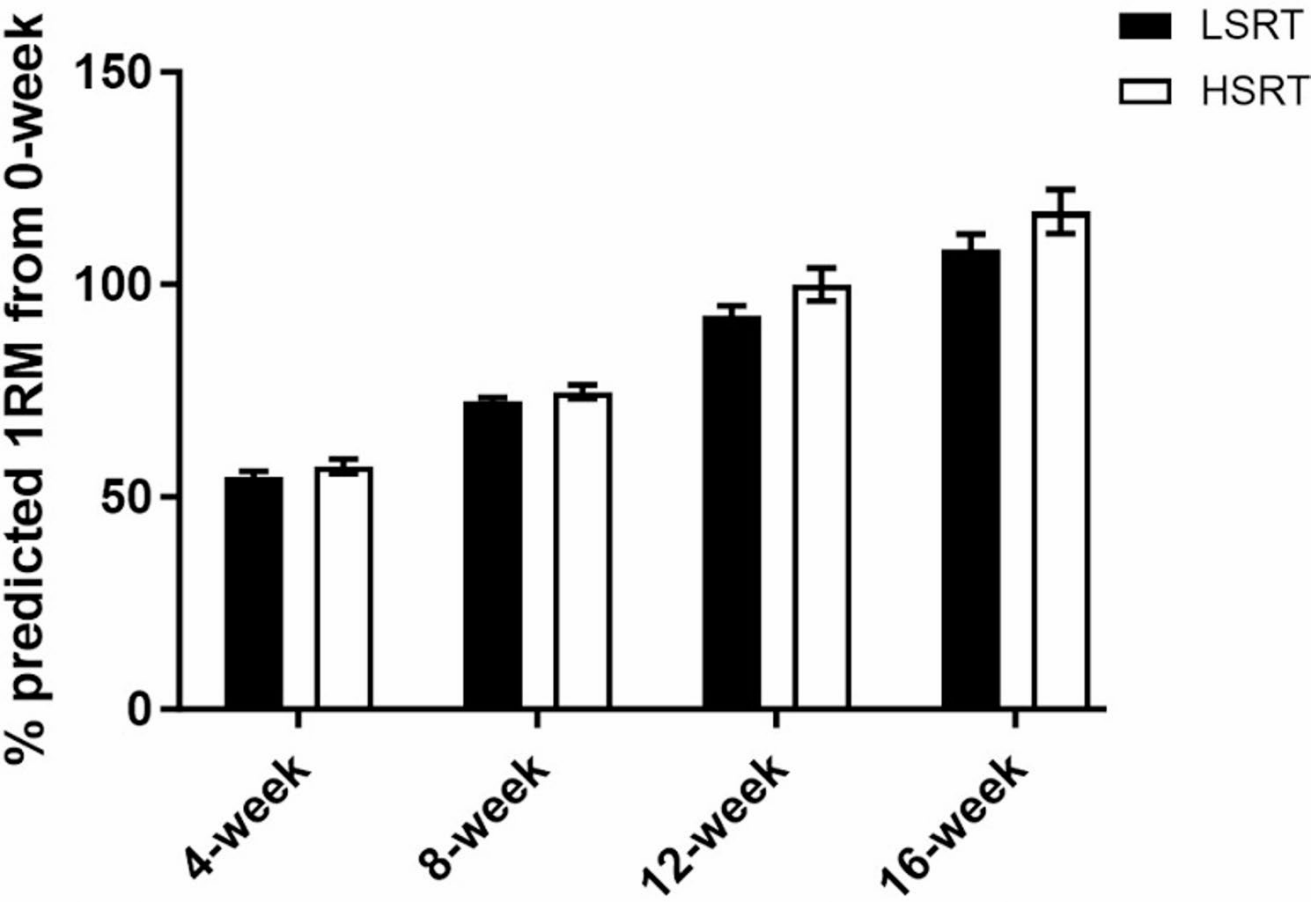
The progression of training intensity as a percentage of the baseline predicted one repetition maximum (1RM). The values were the mean values of five resistance exercises included in the training program. Data were plotted as mean ± SE. HSRT: high-speed resistance training group; LSRT: low-speed resistance training group

One participant in the LSRT group and two in the HSRT group withdrew due to unrelated health issues **(**Fig. 1**)**. Attendance rates were 96.6% and 98.1% in the LSRT and HSRT groups, respectively. Training intensity (mean value of five exercises) progressively increased from 56% of baseline predicted 1RM in the first month to 112% in the last month **(**Fig. 2**)**. There were no differences in exercise intensity between the two training groups.

### Effects of intervention on body composition

**Fig. 3:**
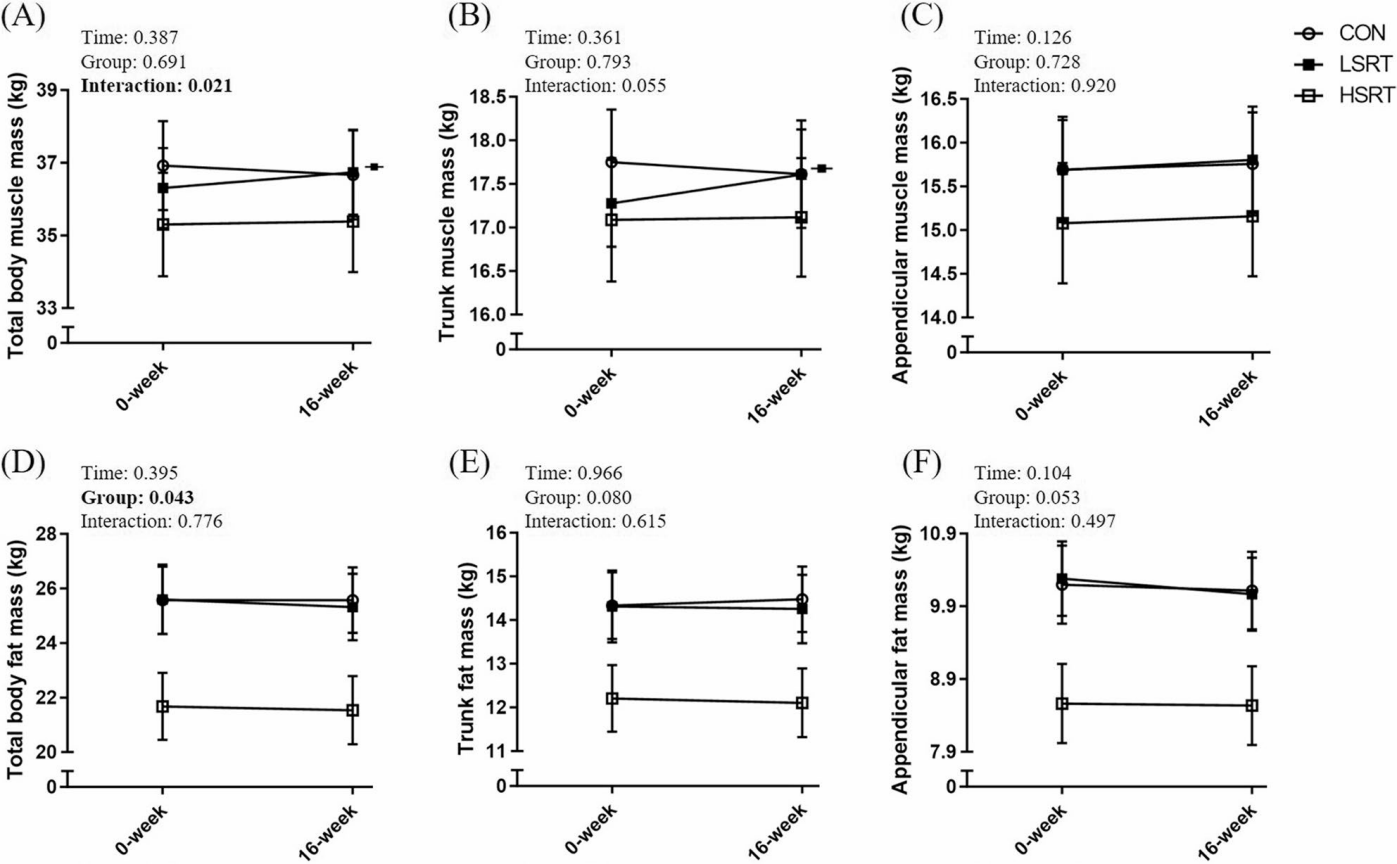
Effects of interventions on total and regional (appendicular and trunk) muscle and fat mass in adults with sarcopenic obesity. Data were plotted as mean ± SE. Symbols denote significant differences from baseline within the corresponding group. CON: control group; HSRT: high-speed resistance training group; LSRT: low-speed resistance training group

There was a significant time-by-group interaction in total body muscle mass (partial η²: 0.105; *p* = 0.021) **(**Fig. 3A**)**. Specifically, only the LSRT group, but not the control and HSRT groups, showed a significant increase in total body muscle mass (the changes of total body muscle mass: control group: -0.70 ± 0.50% (effect size = 0.03); the LSRT group: 1.14 ± 0.44% (effect size = 0.08); the HSRT group: 0.35 ± 0.51% (effect size = 0.01)). There was a trend of time-by-group interaction in trunk muscle mass among groups (*p* = 0.055) **(**Fig. 3B**)**. Only the LSRT group, but not the control and HSRT groups, showed a significant increase in trunk muscle mass (the changes of trunk muscle mass: control group: -0.79 ± 0.77% (effect size = 0.04); the LSRT group: 2.00 ± 0.83% (effect size = 0.13); the HSRT group: 0.36 ± 0.80% (effect size = 0.01)). There was no time-by-group interaction in appendicular muscle mass (*p* = 0.920) **(**Fig. 3C**)**. Regarding the fat mass, there was no time-by-group interaction in appendicular fat mass, trunk fat mass, and total body fat mass (*p* > 0.05) **(**Fig. 3D-F**)**.

### Effects of intervention on physical function

**Fig. 4:**
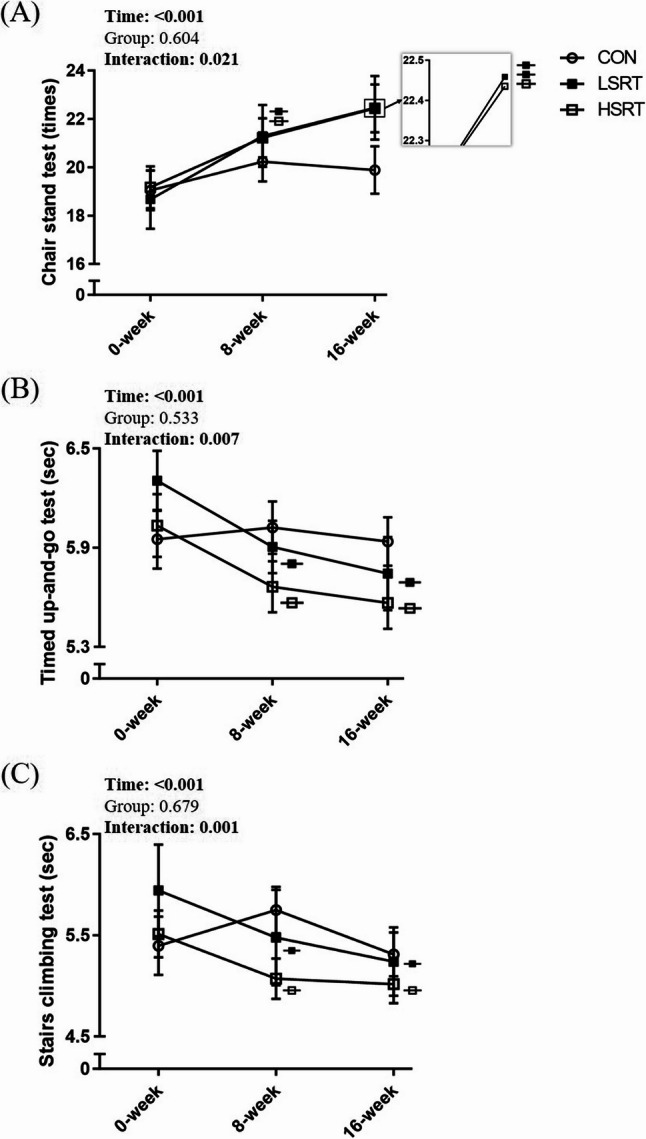
Effects of interventions on chair stand test (A), timed up-and-go test (B), and stairs climbing test (C) in adults with sarcopenic obesity. Data were plotted as mean ± SE. Symbols denote significant differences from baseline within the corresponding group. Two identical symbols indicate significant differences between the 16-week and 8-week assessments within the same group. CON: control group; HSRT: high-speed resistance training group; LSRT: low-speed resistance training group

Significant time-by-group interactions were observed in CST (partial η²: 0.116; *p* = 0.021) **(**Fig. 4A**)**, TUG (partial η²: 0.119; *p* = 0.007) **(**Fig. 4B**)**, and SCT (partial η²: 0.089; *p* = 0.001) **(**Fig. 4C**)**. Compared to the control group, both the LSRT and HSRT groups demonstrated significant improvements in all physical function measures at both 8 and 16 weeks. In the LSRT group, CST performance improved by 16.9% at week 8 (effect size = 0.53) and further increased at week 16 (effect size = 0.61). TUG performance improved by 5.8% at week 8 (effect size = 0.48) and remained improved at week 16 (effect size = 0.57). Similarly, SCT performance improved by 7.7% at week 8 (effect size = 0.21) and this improvement was sustained at week 16 (effect size = 0.36). In the HSRT group, CST performance improved by 12.6%, TUG by 5.6%, and SCT by 7.1% at week 8 (effect sizes were 0.51, 0.45, and 0.43, respectively), and these improvements were maintained at week 16 (effect size = 0.73, 0.56, and 0.49).

### Effects of intervention on salivary hormones

**Fig. 5:**
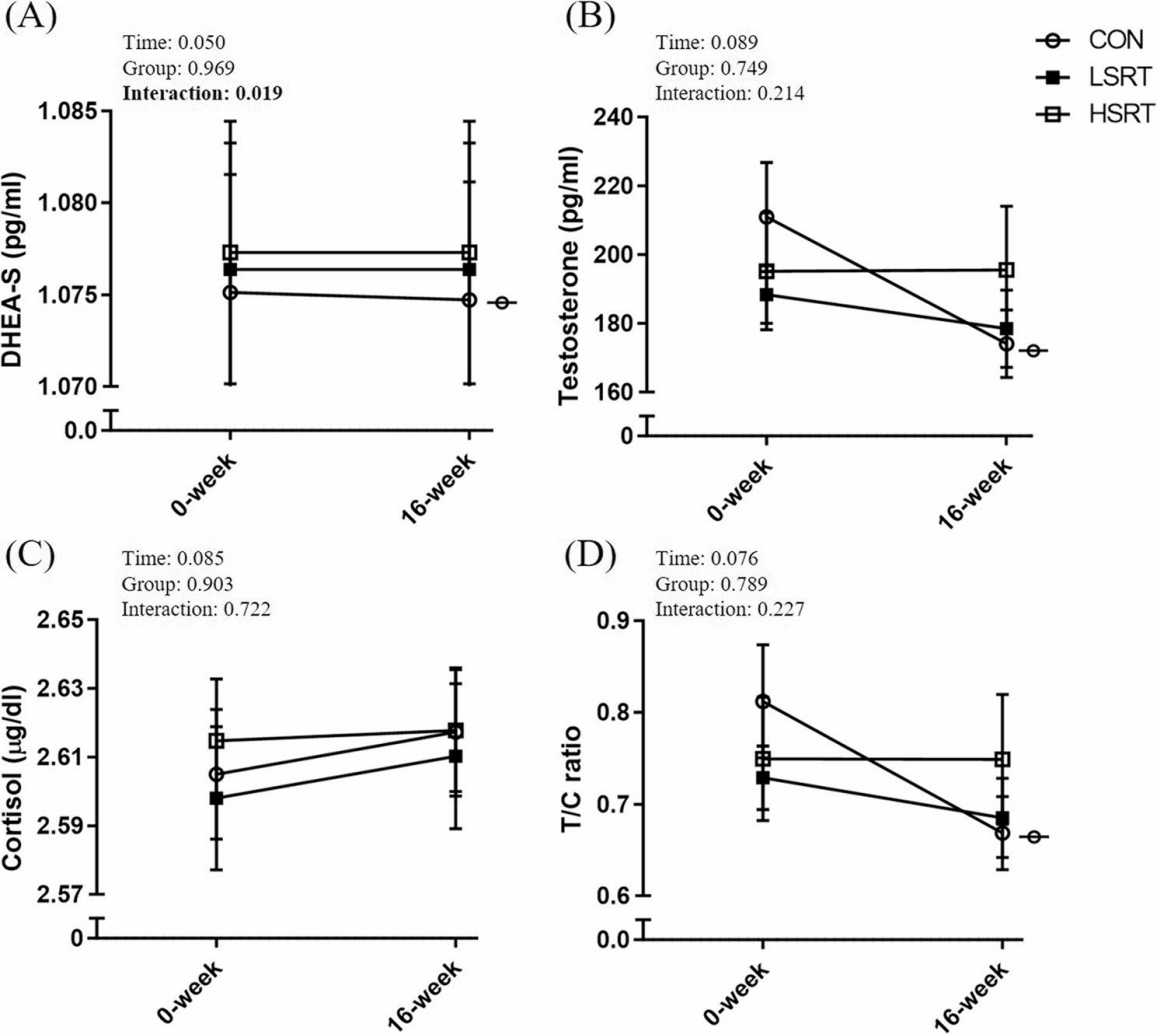
Effects of interventions on salivary dehydroepiandrosterone sulfate (DHEA-S) (A), testosterone (B), cortisol (C), and the testosterone-to-cortisol (T/C) ratio (D) in adults with sarcopenic obesity. Data were plotted as mean ± SE. Symbols denote significant differences from baseline within the corresponding group. CON: control group; HSRT: high-speed resistance training group; LSRT: low-speed resistance training group

Significant time-by-group interactions were only observed in DHEA-S (partial η²: 0.107; *p* = 0.019). Specifically, only the control group, but not the LSRT and HSRT groups, showed a significant decrease in DHEA-S (effect size = 0.01) **(**Fig. 5A**)**. In the control group, the absolute change in DHEA-S was − 0.4 pg/L (95% CI: -0.79 to -0.02 pg/L), representing a -0.04% change (95% CI: -0.07% to -0.002%). There were no significant time-by-group interactions in testosterone **(**Fig. 5B**)**, cortisol **(**Fig. 5C**)**, and the testosterone to cortisol (T/C) ratio (*p* > 0.05) **(**Fig. 5D**)**. Within-group comparisons showed that testosterone and the T/C ratio decreased only in the control group, where testosterone decreased 2.47 ± 9.11% (effect size = 0.55) and the T/C ratio decreased 3.03 ± 8.86% (effect size = 0.54). No significant changes were observed in salivary testosterone, DHEA-S, cortisol, or the T/C ratio in either the LSRT or HSRT group from baseline to post-intervention (*p* > 0.05).

## Discussion

Understanding the distinct effects of LSRT and HSRT on body composition, physical function, and hormone levels in adults with SO is essential for developing effective interventions. We found that both LSRT and HSRT improved physical function in adults with SO. In the LSRT group, a trivial increase in muscle mass was observed. Regarding hormonal outcomes, levels of testosterone, cortisol, and the T/C ratio remained unchanged across groups, while DHEA-S declined only in the control group, suggesting that resistance training may help maintain this anabolic marker in the short term.

Traditionally, LSRT has been recommended for older adults due to safety considerations. Previous studies have shown that LSRT increases total body muscle mass by 5.2% (assessed by BIA) and 2.7% (assessed by DXA) in individuals with SO, where sarcopenia was defined using ASM/Ht² and obesity using BMI [41, 50]. A meta-analysis similarly reported that 6 to 54 weeks of LSRT increased total body muscle mass by 3.1% (measured by DXA) in postmenopausal women [51]. In line with these findings, our study demonstrated a modest increase in total muscle mass in the LSRT group (1.14%). Although the increase was modest, given that muscle mass typically declines by 1–2% per year after the age of 50 [1], such improvement may help attenuate age-related muscle loss. Nevertheless, the improvement observed in the LSRT group was absent in the HSRT group. This contrasts with findings of Polo-Ferrero et al., who reported that HSRT significantly increased ASM by 8.4% (as measured by BIA) in women with SO [52]. The difference between our findings and those of Polo-Ferrero et al. is likely due to the longer intervention period and different body composition assessments in their study, which lasted 8 months and used BIA, whereas ours was a 4-month program using DXA. The extended training period in their study likely contributed, at least in part, to the larger muscle mass gains observed [52].

Among studies that have directly compared LSRT and HSRT, results have been inconsistent. Henwood et al. found that both LSRT and HSRT increased total body muscle mass by 3.7% and 2.8% (measured by DXA) in the elderly [28], whereas Yoon et al. reported that only HSRT, not LSRT, led to gains in muscle mass (4.2% measured by BIA) [48]. These discrepancies may be attributed to differences in training protocols. In our study, both LSRT and HSRT were performed for 16 weeks using 3 sets of 10 repetitions at 70% of predicted 1RM. In contrast, Henwood et al. used a 24-week protocol in which the HSRT group trained at a lower intensity than the LSRT group, but with the same number of repetitions. Yoon et al. used a 12-week program in which the HSRT group trained at a lower resistance but higher repetitions than the LSRT group. These variations in intensity, the number of repetitions, and duration may explain the inconsistent outcomes.

Our finding of increased total body mass in the LSRT group but not the HSRT group aligns with prior evidence from acute exercise studies, which have shown that low-speed muscle contractions—characterized by greater time under tension—lead to greater lactate accumulation and protein synthesis than high-speed muscle contractions [38, 39]. Collectively, our findings, along with prior evidence, suggest that when the number of repetitions is matched, LSRT may be more effective than HSRT in increasing muscle mass in individuals with SO.

Interestingly, the increase in muscle mass observed in the LSRT group was primarily attributable to gains in trunk muscle mass. Most resistance training studies report total or appendicular muscle mass, and few specifically examine changes in trunk muscle mass [53–55]. Although our exercises did not directly target the trunk, all training was performed in a standing position, requiring postural stabilization. Trunk musculature plays a central role in maintaining stability during limb movement [56]. Throughout training, participants were consistently instructed to maintain an upright posture with the chest lifted and abdomen braced, which likely led to continuous, unintentional activation of the trunk muscles across repetitions and sessions. This persistent stabilizing demand may have been sufficient to stimulate hypertrophy in trunk musculature, contributing to the observed increases.

To our surprise, our training, which targeted both upper- and lower-limb muscle groups, did not result in a significant increase of ASM in both LSRT and HSRT groups. This finding is different from previous publications [52, 57]. One possible explanation is related to the duration of the intervention. For example, Lee et al. reported no increase in ASM in individuals with SO after 3 months of intervention [58]. By contrast, Polo-Ferrero et al. observed increases in ASM in participants with SO after an 8-month intervention, whereas our intervention lasted only 4 months [52]. These findings suggest that longer training periods would elicit more detectable hypertrophy in limb musculature.

Although muscle mass increased in the LSRT group, salivary testosterone, DHEA-S, and cortisol levels, as well as the T/C ratio, did not change in either training group. This finding is consistent with previous studies, which have shown that muscle gains after resistance training do not always coincide with increases in resting anabolic hormones, particularly in older adults and women [54, 55, 59, 60]. Testosterone responses to resistance training are often limited in women, possibly due to the absence of Leydig cells, which are present in men and contribute to testosterone production [61]. The predominance of female participants (86%) in our study may partly explain the lack of changes in testosterone. Additionally, age-related declines in hormone levels and reduced endocrine responsiveness likely contributed where studies have shown that aging is associated with lower resting testosterone levels and blunted adaptations following resistance training [62, 63]. Similarly, although DHEA-S levels increase with training in young adults [19, 64], such effects are less evident in older populations [54]. In the control group, a slight reduction in DHEA-S was observed. However, the change was very minimal. This minor decline may reflect the early stages of normal age-related hormonal decrease, which could become more pronounced over a longer period [65]. Collectively, these results suggest that LSRT and HSRT may help maintain DHEA-S levels over the short term, despite not increasing absolute levels.

In this study, both LSRT and HSRT improved physical function in adults with SO after 8 weeks, with no significant difference between the two protocols. Previous studies also reported improvement in physical function among individuals with SO following LSRT or HSRT [52, 58]; however, these studies did not directly compare the relative effectiveness of the two training modes. Studies directly comparing LSRT and HSRT in other populations have reported mixed findings, likely due to differences in intervention duration and participant characteristics. Some studies have found HSRT to be more effective in improving TUG, SCT, and CST, especially in sedentary or high-fall-risk populations [28, 31, 65]. In contrast, others have shown similar benefits from both LSRT and HSRT with 12–24 weeks of intervention [28, 30]. In the current study, participants had relatively good baseline function, which may explain the comparable effects. Taken together, the potential advantage of HSRT over LSRT in improving physical function may be more evident in individuals with greater functional impairment.

Beyond physical function, several studies using muscle biopsies have investigated the effects of RT at different contraction velocities on muscle fiber types and their functional properties. Evidence suggests that regardless of contraction velocity, RT generally increases the cross-sectional area (CSA), contractile force, and power output of type II muscle fibers, specifically type IIA and IIX [66–68]. Regarding changes in myosin heavy chain (MHC) isoform distribution, both high-velocity and low-velocity contractions have been shown to increase the proportion of MHC IIA, indicating an increased prevalence of hybrid phenotypes [67–69]. However, findings regarding MHC IIX proportions remain equivocal. While Schuenke et al. observed a reduction in MHC IIX percentages across both high- and low-speed protocols [68], Pareja-Blanco et al. found that only training with greater velocity loss (slower, more fatigued training) led to a significant decrease in MHC IIX content, whereas maintaining high velocities preserved this fast-twitch phenotype [67]. In summary, cellular-level research indicates that RT consistently enhances type II muscle fiber size and increases MHC IIA percentages irrespective of velocity; however, its effect on reducing MHC IIX proportions remains inconsistent. It is noteworthy that current literature on this topic primarily focuses on younger populations [66–69]. Furthermore, experimental designs often involve confounding variables, as training intensities or repetitions frequently differ between velocity group [67–69]. Given the limited relevant research on individuals with SO, future studies using muscle biopsies and mechanistic assessments are warranted.

This study has some limitations. First, the assessor for physical function was not blinded; however, all assessment procedures were standardized prior to the study to minimize potential bias. Second, the sample size was relatively small, which may limit the statistical power of our findings. Third, we did not perform sex-based analyses because the majority of participants were women. Fourth, the criteria used to define obesity and sarcopenia were based on Taiwan’s criteria and Asian criteria, respectively, which may limit the generalizability of our findings to other populations. Lastly, participants’ dietary intake, supplement use, and medications were not strictly controlled, which may have influenced the results. Nevertheless, the randomized controlled trial design helps to minimize the influence of such confounding factors.

In conclusion, both low-speed and high-speed resistance training improved physical function and may help maintain DHEA-S levels in adults with SO. Although the increase in muscle mass observed with LSRT was trivial, this training approach may still contribute to supporting muscle health. Our findings provide practical guidance for designing tailored resistance training programs for adults with SO.

## Data Availability

The datasets generated during and/or analyzed during the current study are available from the corresponding author on reasonable request.
